# High-resolution data and maps of material stock, population, and employment in Austria from 1985 to 2018

**DOI:** 10.1016/j.dib.2023.108997

**Published:** 2023-02-20

**Authors:** Franz Schug, Dominik Wiedenhofer, Helmut Haberl, David Frantz, Doris Virág, Sebastian van der Linden, Patrick Hostert

**Affiliations:** a Geography Department, Humboldt-Universität zu Berlin, Unter den Linden 6, Berlin 10099, Germany; b Integrative Research Institute on Transformations of Human-Environment Systems, Humboldt-Universität zu Berlin, Unter den Linden 6, Berlin 10099, Germany; c SILVIS Lab, Department of Forest and Wildlife Ecology, University of Wisconsin-Madison, 1630 Linden Dr., Madison, WI 53706, USA; d Institute of Social Ecology, University of Natural Resources and Life Sciences Vienna, Schottenfeldgasse 29, Vienna 1070, Austria; e Geoinformatics – Spatial Data Science, Trier University, Behringstraße 21, Trier 54296, Germany; f Institute of Geography and Geology, University of Greifswald, Friedrich-Ludwig-Jahnstraße 18, Greifswald 17489, Germany

**Keywords:** Buildings, Change aftereffect trend analysis, Industrial ecology, Infrastructure, Landsat, Socio-economic metabolism, Time series analysis

## Abstract

High-resolution maps of material stocks in buildings and infrastructures are of key importance for studies of societal resource use (social metabolism, circular economy, secondary resource potentials) as well as for transport studies and land system science. So far, such maps were only available for specific years but not in time series. Even for single years, data covering entire countries with high resolution, or using remote-sensing data are rare. Instead, they often have local extent (e.g., [1]), are lower resolution (e.g., [2]), or are based on other geospatial data (e.g., [3]). We here present data on the material stocks in three types of buildings (commercial and industrial, single- and multifamily houses) and three types of infrastructures (roads, railways, other infrastructures) for a 33-year time series for Austria at a spatial resolution of 30 m. The article also presents data on population and employment in Austria for the same time period, at the same spatial resolution. Data were derived with the same method applied in a recent study for Germany [4].

**Specifications Table**

**Table:** 

Subject	Earth and Planetary Sciences

Specific subject area	Remote Sensing, Industrial Ecology
Type of data	Raster (GeoTIFF)
How the data were acquired	Raster data of the nationwide distribution of material stocks, population, and employment in Austria from 1985 to 2018 required algorithmic analysis using a combination of three existing datasets: (a) a spatially explicit reference map of material stocks and building volume for the study area for 2018, previously generated in Haberl et al., 2021 [5] (b) high-resolution Landsat satellite images for the complete study period (c) census population and employment data for Austria, retrieved from the Austrian Federal Statistics Office (*Statistik Austria*)
Data format	Raw
Description of data collection	All underlying data are freely and openly available for download (see section on data source location).
Data source location	The spatially explicit reference map of material stocks and building volume for the study area for 2018 is available in a data repository: • Haberl, H. et al.: High-Resolution Maps of Material Stocks in Buildings and Infrastructures in Austria and Germany, Environ. Sci. Technol. 2021, 55, 5, doi:10.1021/acs.est.0c05642. Data available here: 10.5281/zenodo.4522892 High-resolution Landsat satellite imagery is available for download in the Landsat image archive: • https://earthexplorer.usgs.gov/ Census population and employment data for Austria is available from Statistik Austria: • Institution: Statistik Austria (official authority) • City: Vienna • Country: Austria • Address: Guglgasse 13, A-1110 Vienna • Population data (requires guest login): statcube.at/statistik.at/ext/statcube/jsf/tableView/tableView.xhtml • Employment data: https://www.statistik.at/fileadmin/pages/257/3_Erwerbstaetigkeit_Zeitreihen_bis2021.ods Census population and employment data for Austria as used for this study is also available in the Supplementary Materials (states_pop_AT.txt, states_jobs_AT.txt) and in a data repository: • Repository name: Zenodo • Data identification number: 10.5281/zenodo.7373398 • Direct URL to data: https://zenodo.org/record/7373398
Data accessibility	Raster data of the nationwide distribution of material stocks, population, and employment in Austria from 1985 to 2018 are publicly available for download in a data repository: • Repository name: Zenodo • Data identification number: 10.5281/zenodo.7195101 • Direct URL to data: https://zenodo.org/record/7195101 Tabular data used to create Figures 1 to 4 as well as population and employment data extracted from Statistik Austria are available as Supplementary Material of the article and in a data repository: • Repository name: Zenodo • Data identification number: 10.5281/zenodo.7373398 • Direct URL to data: https://zenodo.org/record/7373398

## Value of the Data

• High-resolution maps of changes in material stocks in buildings and infrastructures as well as employment and population are required in many scholarly contexts and practice systems, as well as in Spatial Planning.
• Researchers in Industrial Ecology, Social Ecology, Integrated Land System Science, Demography, Geography and other fields benefit, as well as practitioners e.g. in Land Use and Spatial Planning, Energy and Transport Policy, Economic and Social Policy.
• Data can serve as inputs for mapping and modelling of (a) availability of secondary resources (urban mining), (b) energy demand (e.g. space heating, transport energy), (c) resource requirements for maintenance (e.g. refurbishment of buildings, road surfaces).

## Objective

High-resolution spatial data on the distribution of material stocks were created for Germany and Austria for 2018 based on remote sensing and other geodata in a previous study [5,6]. Subsequently, annual historic data of material stocks, population and employment for Germany from 1985 to 2018 were generated based on this reference dataset [4]. However, spatially explicit time series data at high resolution have not yet been created for Austria. Here, we transfer the approach presented in Schug et al. [4] and create annual historic data of material stocks, population and employment for Austria from 1985 to 2018. While a proof of concept of the workflow has been established before, high-resolution data for other countries allow researchers to use a consistent time series information on three key indicators of the socio-economic metabolism for applied research in their region. In Austria, for example, the long-term socio-ecological research platforms (*Eisenwurzen* and Tyrolean High Alps [7]) will benefit from the availability of high-resolution historic data on the socio-economic metabolism. Austria is a country of considerable landscape diversity, featuring High Alps in the Western part of the country and flatter landscapes with agricultural land uses in the North, East, and South. Agglomerations tend to be, but are not uniquely, located in the lower areas of the country. We provide raw raster data, but also data descriptions and aggregations that can be a direct input to research and applications in numerous fields. While the according publication [4] establishes a proof-of-concept, this transfer and the data we present here are a valuable spatial extension of the approach.

## 1 Data Description

**Fig. 1:**
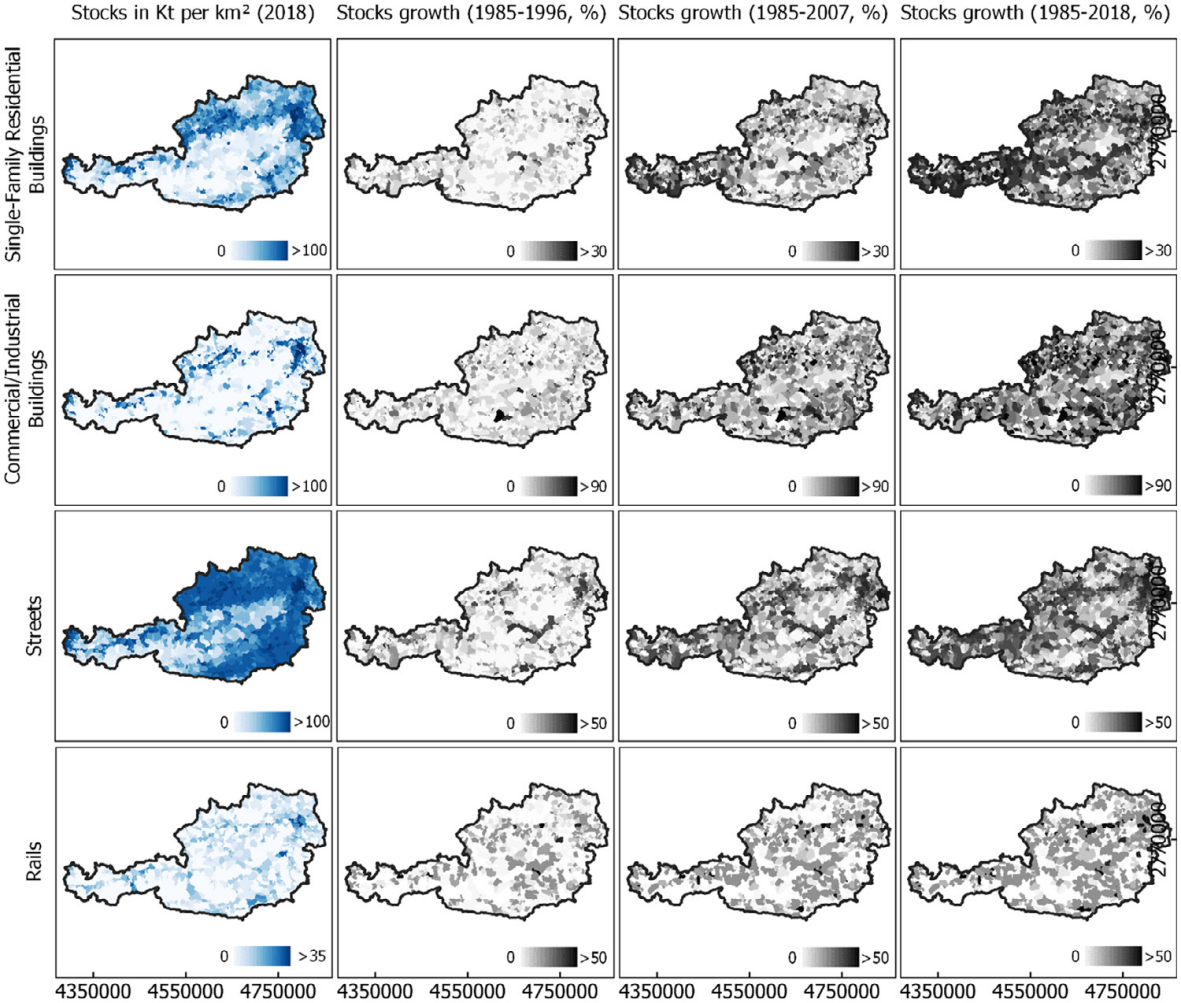
Material stock density (Kt/km²) in 2018 and total growth (%) in three selected periods in Austria in four example categories (single-family buildings, commercial and industrial buildings, roads and rails). Data for 2018 from [5]. All other years created for this dataset. Annual data were temporally aggregated to regular time steps of 11 years for illustration. For illustration purposes, pixel-wise data were spatially aggregated to the level of municipalities. See Supplementary Materials for data to create Fig. 1, files stocks_buildingsingle_AT.csv, stocks_buildingcomm_AT.csv, stocks_street_AT.csv, stocks_rail_AT.csv.

**Fig. 2:**
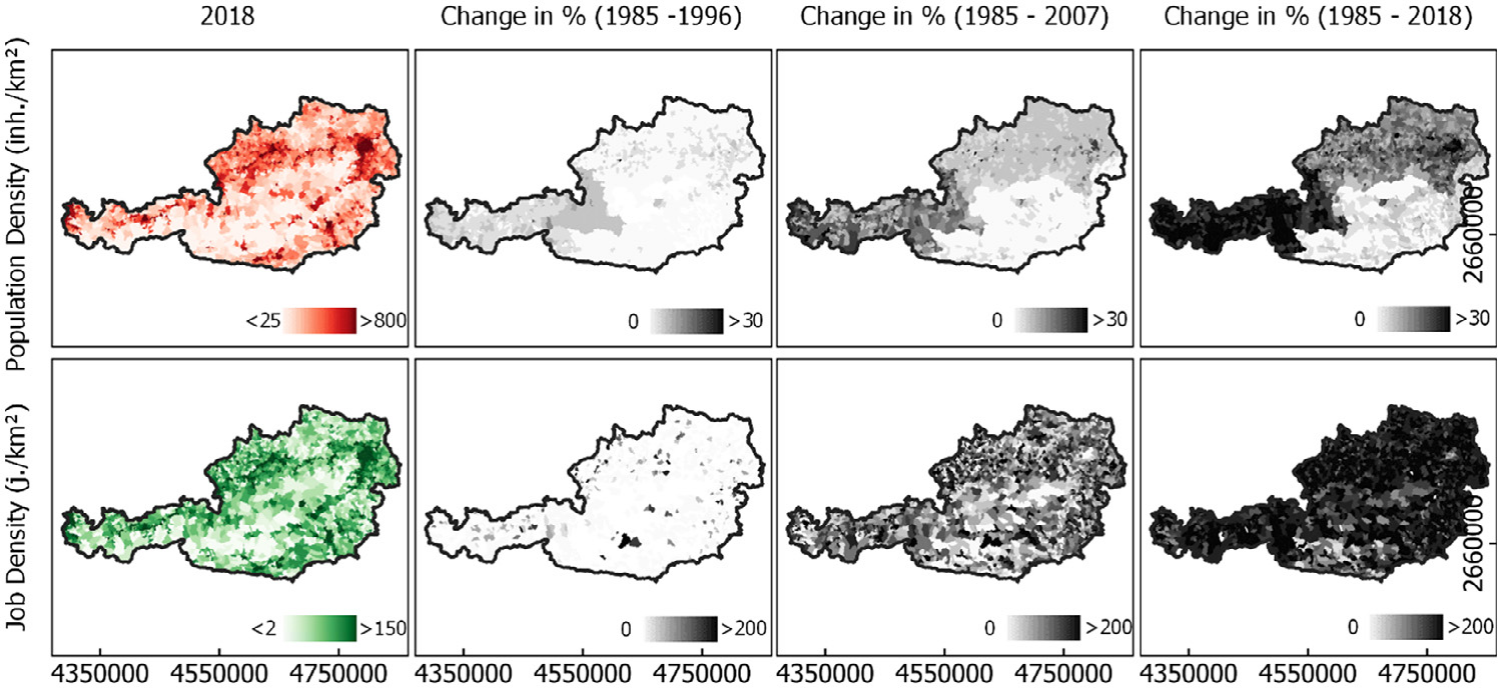
Population density (cap./km²) and job density (jobs/km²) in 2018 and total changes (%) in three selected periods in Austrian municipalities. Data generated for this dataset based on a methodology described in [4]. Annual data were temporally aggregated to regular time steps of 11 years for illustration. For illustration purposes, pixel-wise data were spatially aggregated to the level of municipalities. See Supplementary Materials for data to create Fig. 2, files pop_AT.csv, jobs_AT.csv.

**Fig. 3:**
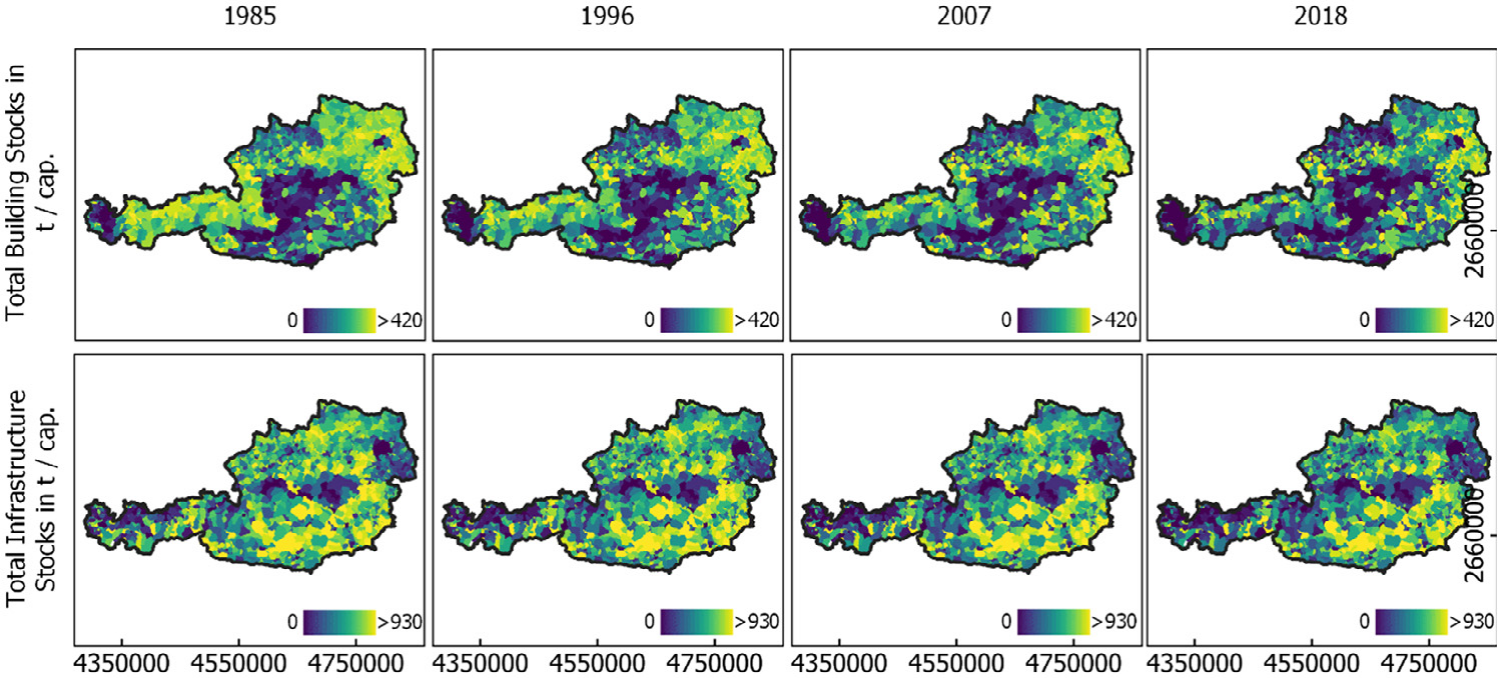
Material stocks per capita in buildings (top) and infrastructure (bottom) per municipality in four different years. Annual data were temporally aggregated to regular time steps of 11 years for illustration. For illustration purposes, pixel-wise data were spatially aggregated to the level of municipalities. See Supplementary Materials for data to create Fig. 3, stocks_buildingtotal_AT.csv, stocks_infratotal_AT.csv, pop_AT.csv.

**Fig. 4:**
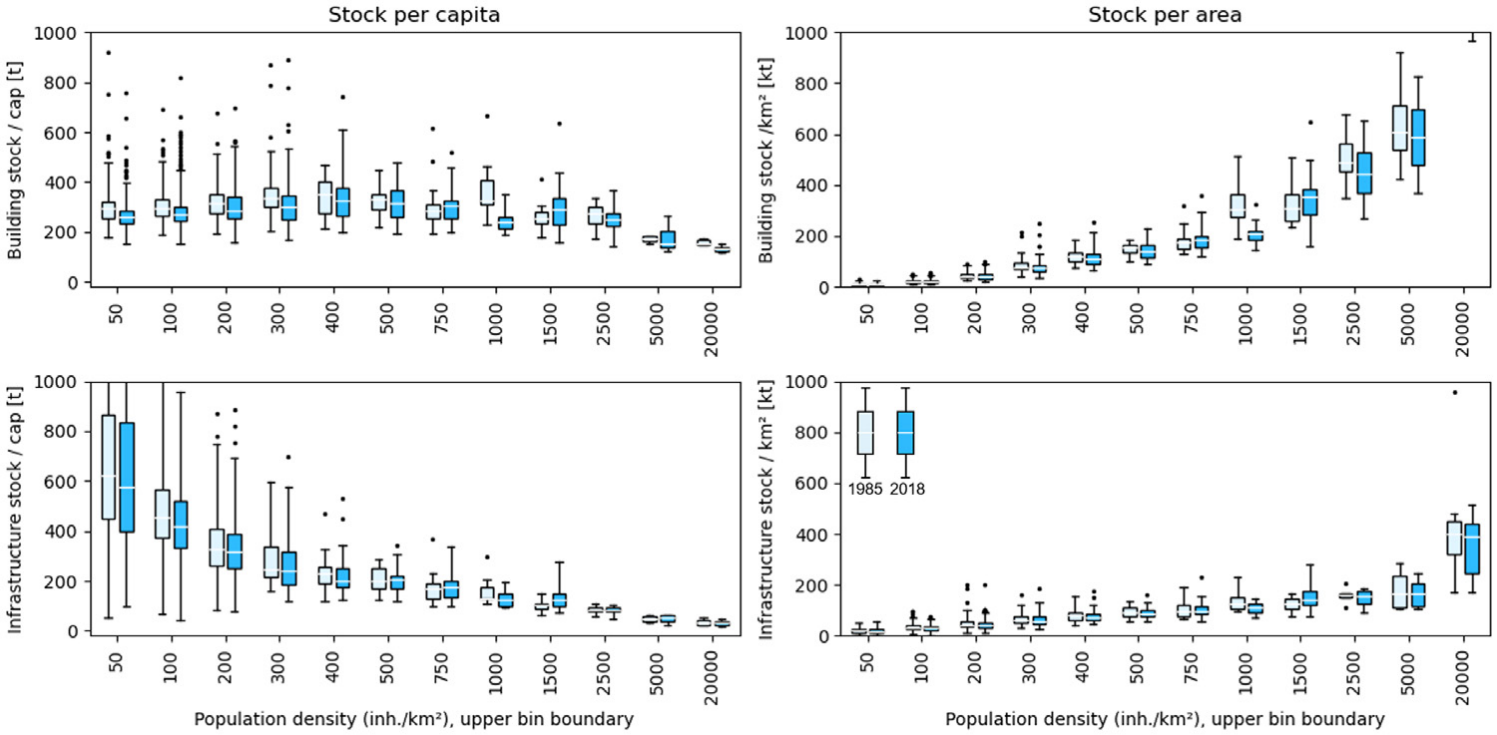
Building (top) and infrastructure (bottom) stocks per capita (left) and per area (right) in Austria. Boxplots represent stock values of all municipalities in 1985 and 2018. Population density (x-axis) represents the upper boundary of the boxplot bins, e.g. 500 for all municipalities with a population density between 400 and 500. X-axes of the four plots used varying bin sizes in order to allow for a better distribution of data within the bins. See Supplementary Materials for data to create Fig. 4, files stocks_buildingtotal_AT.csv, stocks_infratotal_AT.csv, pop_AT.csv.

This article describes raster-based nation-wide annual data on material stocks, population, and employment for Austria from 1985 to 2018 with a spatial resolution of 30 m. The datasets represent the development of three key parameters of the socio-economic metabolism. The data include raster-data about the per-pixel amount of the mass of materials in two selected categories of built structures, i.e., buildings and infrastructures. Six sub-categories are distinguished for each year between 1985 and 2018. Two additional datasets provide the per-pixel number of people and workplaces for the same years with the same spatial resolution (Table 1). Supplementary materials of this article provide aggregated data of these raster data on a municipality level used to create Fig. 1, Fig. 2, Fig. 3, Fig. 4, as well as a topographic and political map of Austria.

**Table 1:** All datasets provided in the linked repository. All data are raster data with a spatial resolution of 30 m and represent the mass in tons for material stocks and number of people for population and employment data. In parentheses: According file name of the respective raster dataset in the linked repository.

Raster data, Level 1	Raster data, Level 2	Raster data, Level 3
Total material stocks in buildings and infrastructure (MASS_TOTAL.tif)	Material stocks in all buildings (MASS_BLD_TOTAL.tif)	Material stocks in commercial and industrial buildings (MASS_BLD_COMM.tif)
		Material stocks in multi-family residential buildings (MASS_BLD_MULTI.tif)
		Material stocks in single-family residential buildings (MASS_BLD_SINGLE.tif)
		Material stocks in lightweight buildings (MASS_BLD_LIGHT.tif)
		Material stocks in high-rise buildings (MASS_BLD_HIGH.tif)
	Material stocks in all infrastructure (add material stocks in road, rail, and other infrastructure)	Material stocks in road infrastructure (MASS_STREET.tif)
		Material stocks in rail infrastructure (MASS_RAIL.tif)
		Material stocks in other infrastructure (MASS_OTHER_TOTAL.tif)
Employment (JOBS.tif)		
Population (POPULATION.tif)

### 1.1 Data Structure and Format

The raster datasets in the linked repository are organized along Austrian federal states. For each federal state (abbreviations in states_AT.txt), all raster data come in tiles of 30 × 30 km, corresponding to 1000 × 1000 pixels. The projection is ETRS89-extended / LAEA Europe (EPSG: 3035). All data is provided as compressed GeoTiff (*.tif) files. A virtual data mosaic (*.vrt) is available for all datasets. These files can be opened with all Geographic Information systems that support formats as defined by the Geospatial Data Abstraction Library (GDAL, https://gdal.org). A list of software currently supporting GDAL can be found here: https://gdal.org/software_using_gdal.html#software-using-gdal). All raster data feature 34 bands, where each band represents data from one year (with band 1 = 1985 and band 34= 2018). The data in the supplementary materials include a shape file (.shp) of Austrian municipalities, as well as annual tabular datasets on selected material stocks categories, population, and employment. These data were aggregated from a raster level to a municipality level and used to illustrate data in Figs. 1 to 4 (see figure captions). All tabular data is provided in a .csv format.

### 1.2 Material Stocks

Fig. 1 illustrates the density and relative development of stocks in municipalities in four example categories. Material stock in single-family housing is particularly dense around major agglomerations but is spread throughout the country where topography allows. Development is strong along two major axes in East-West direction and around agglomerations in the North and East, as well as in the state of Tyrol. The growth of material stocks in commercial and industrial buildings is more scattered, with growth rates of more than 90% in selected places from 1985 to 2018. Austria has a rather dense distribution of material stocks in streets in its non-Alpine regions, with one axis in East-West direction cutting through the mountainous West of the country. Material stocks in rails are considerably lower, with no clear spatial pattern of development. From 1985 to 2018, material stock in different building and infrastructure types grew at different rates. Stocks in single-family buildings grew from 0.97 to 1.02 Gt (+ ∼5%), in multi-family buildings from 0.56 to 0.61 Gt (+ ∼9%), in commercial and industrial buildings from 0.47 to 0.62 Gt (+ ∼32%), in road infrastructure from 1.99 to 2.05 Gt (+ ∼3%), in rail infrastructure from 78 to 83 Mt (+ ∼6%) and in other infrastructure from 87 to 110 Mt (+ ∼26%).

### 1.3 Population and Employment

This dataset features population and employment on a pixel level, with 30 m spatial resolution. Both population and jobs are distributed in and around agglomerations as well as along two major axes of development (East-West and East-South-West). The latter are primarily defined by the topography of Austria as an Alpine country. Population growth is shaped by large differences amongst federal states. Municipalities in Western (i.e., Tyrol, Vorarlberg, Salzburg) and Northern (i.e., Upper Austria, Lower Austria, Vienna) Austria have a comparably high population growth rate. The lowest increase in population density was found in Styria and Carinthia. Job density follows population density (see Supplementary Material for state locations). The growth of employment is distributed more evenly, with possible rates of 200% and more. Growth is not bound to agglomerations or specific regions. Fig. 2 illustrates population and employment, aggregated on a municipality level.

### 1.4 Material Stocks and Population

Fig. 3 illustrates material stock quantity per capita for building and infrastructure stocks on an aggregated municipality level. Large stocks per capita can be observed in Western, Northern and Southern rural parts of the country, with an overall building stock per capita of ca. 261 t and an overall infrastructure stock per capita of 281 t in 1985. In 2018, those numbers slightly decreased to 254 t and 253 t respectively in 2018. Agglomerations and Alpine regions show considerably lower values, with some Alpine municipalities featuring high infrastructure stocks.

Fig. 4 illustrates building and infrastructure stocks per capita in 1985 and 2018 across all municipalities. Municipalities are organized in bins according to their population density. Building and infrastructure stocks per capita were slightly falling from 1985 to 2018. While there is an effect of substantially decreasing infrastructure stocks per capita where population density in the respective municipality rises, this effect cannot be observed for building stocks. Per unit area, building stocks are rapidly increasing with increasing population density. However, this increase is notably less pronounced for infrastructure stocks. The lowest observed stocks per capita across all municipalities in 2018 are ∼175 t/cap for buildings and ∼50 t/cap for infrastructure.

## 2 Experimental Design, Material and Methods

The data presented in this data article are annual high-resolution raster data on material stocks from 1985 to 2018. They were generated based on an existing reference dataset for 2018 provided by Haberl et al. [5]. There, high-resolution maps, i.e., with a pixel resolution of 10 m, of material stocks featuring a large diversity of material types were created for the countries of Austria and Germany. Data were derived by combining information on built-up density, building height, and building type with material intensity factors that represent a typical mass and material composition per cubic meter of a building or square meter of a specific type of infrastructure. Material intensities were retrieved from the literature ([8], [9], [10], [11], [12], [13], [14], [15] as in [5]). Modelling of built-up density and building height in this previous study are discussed in Schug et al. [16] and Frantz et al. [17]; therein, intra-annual time series of Copernicus Sentinel-1 [18] and Sentinel-2 data [19] were analysed using machine learning regression methods. Building type data, i.e., data that distinguishes residential housing from commercial and industrial buildings, were created using the same input data and random forest modelling [20].

**Fig. 5:**
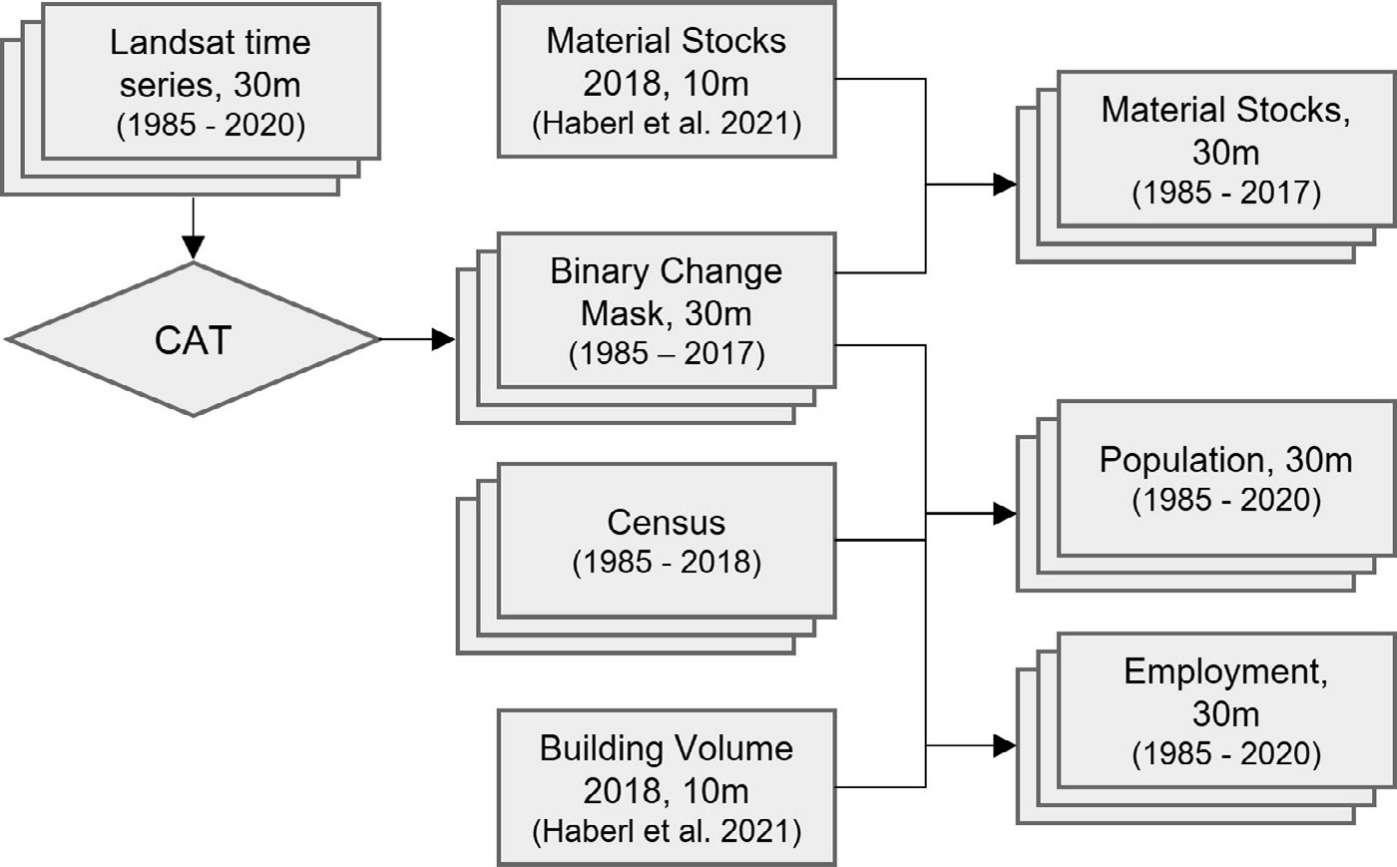
Processing workflow (adapted from [4]) from Landsat time series data, census data and data on material stocks and building volume from a previous study for 2018 to annual historic maps on material stocks, population and employment.

The dataset presented here was created based on previously established data and workflows (Fig. 5), where annual historic data of material stocks and population were generated for Germany [4]. In this study, we transferred this approach to create data for Austria. We first downloaded all freely available [21] Landsat TM, ETM+ and OLI data from 1985 to 2020 for the entire territory of Austria, i.e., 8872 scenes. Data were pre-processed, e.g., atmospherically corrected and cloud-masked, using the Framework for Operational Radiometric Correction for Environmental monitoring (FORCE [22]). We resampled the spatial resolution of material stock data for 2018 from 10 m to 30 m in order to establish a consistency with the spatial resolution of Landsat data. We then used a change-aftereffect trend analysis (CAT [23]) to create annual maps of land surface change from 1985 to 2018. CAT captures gradual (trend) or abrupt (change) non-seasonal landscape changes based on a multi-annual time series of a spectral vegetation index. CAT divides this time series into two sections, pre- and post-change, based on the detected year of abrupt change, assuming that only one change occurred. We assumed that a complete and permanent removal of vegetation in Austria implies stock building activity. The CAT analysis was performed on an annual basis, where all individual Landsat acquisitions were aggregated to yearly measurements by using the maximum value of the Normalized Difference Vegetation Index (NDVI [24]) per year. Thus, the year of change is determined based on an abrupt decrease in maximum NDVI. Material stock data was then iteratively masked using change maps from 2018 to 1985 in order to track stock building development.

CAT analysis was also used to generate annual historic data of population and employment. However, as population and the availability of jobs are not only driven by the mere presence of buildings, these datasets were not based on a time series of material stock. Instead, we used CAT to create annual data of building volume, i.e., a multiplication of building density and building height. Then, we retrieved annual census data on population and employment at a level of federal states from Statistik Austria, the Austrian Federal Statistical Office [25,26]. We then used a dasymetric mapping approach to disaggregate population and employment data [27] from a level of federal states to a pixel level with 30 m resolution for each year using a weighting factor. In our case, annual building volume was used as a weighting layer for disaggregation.

The quality of the generated datasets is challenging to validate because of the lack of historic and high-resolution reference data on the distribution of material stocks and stock types. It can, however, be approximated by assessing the quality of the underlying data used to create the material stocks map for 2018, i.e., building density (Root-Mean-Squared Error RMSE of 19%), building height (RMSE of 3–4 m), and building type (Overall accuracy of 81.4%, [5]). Material stock values using this approach for 2018 compare well to existing studies (with a tendency for higher estimates due to methodological differences, 5), and the quality of detecting change using the CAT algorithm was between 85% and 95% for all years of change in similar environments [4]. It was also found previously that the used approach for population re-redistribution from census data to single pixels features a Mean Absolute Error of 1335 people across all municipalities (R2 = 0.99) in Germany [20]. We find these quality metrics to be very good in the context of remote sensing based mapping, but acknowledge that errors can possibly accumulate in single local spots.

## CRediT authorship contribution statement

**Franz Schug:** Conceptualization, Methodology, Formal analysis, Investigation, Visualization, Data curation, Writing – original draft. **Dominik Wiedenhofer:** Conceptualization, Writing – review & editing. **Helmut Haberl:** Conceptualization, Resources, Supervision, Writing – review & editing. **David Frantz:** Writing – review & editing. **Doris Virág:** Investigation, Data curation, Writing – review & editing. **Sebastian van der Linden:** Writing – review & editing. **Patrick Hostert:** Writing – review & editing.

## Declaration of Competing Interest

The authors declare that they have no known competing financial interests or personal relationships that could have appeared to influence the work reported in this paper.

## Data Availability

High-resolution maps of material stock, population and employment in Austria from 1985 to 2018 (Original data) (Zenodo). High-resolution maps of material stock, population and employment in Austria from 1985 to 2018 - Supplementary Material (Original data) (Zenodo).

## Appendix Supplementary Materials

Image, application 1
