# Supplementary figures and images for: High-resolution data and maps of material stock, population, and employment in Austria from 1985 to 2018
Source: Data Brief. 2023 Feb 20;47:108997. doi: 10.1016/j.dib.2023.108997 (PMC9999155; doi:10.1016/j.dib.2023.108997)

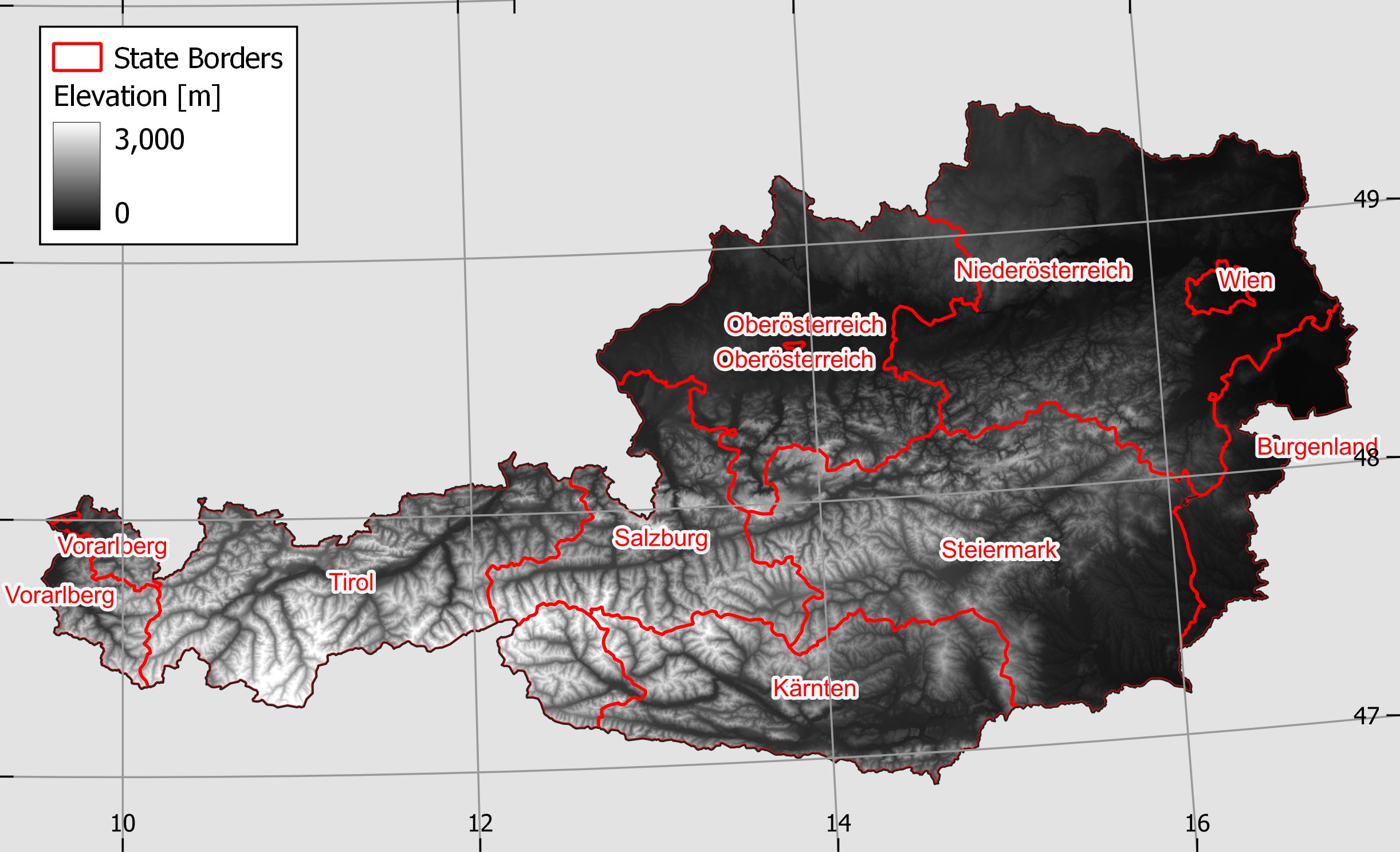

Supplement: Supplementary file 1 [file mmc1.zip › data/Fig_S1.jpg]
